# DNA topoisomerase II*α* expression and the response toprimary chemotherapy in breast cancer

**DOI:** 10.1038/sj.bjc.6601185

**Published:** 2003-08-12

**Authors:** G MacGrogan, P Rudolph, I de Mascarel, L Mauriac, M Durand, A Avril, J M Dilhuydy, J Robert, S Mathoulin-Pélissier, V Picot, A Floquet, G Sierankowski, J M Coindre

**Affiliations:** 1 Departments of Pathology, Oncology, Surgery, Radiotherapy, Biochemistry and Biostatistics, Institut Bergonié, 229 Cours de l'Argonne, 33076 Bordeaux cedex France; 2 Department of Pathology and Lymph Node Registry appointed by the German Association of Pathologists, University of Kiel, Department of Pathology, Michaelisstr. 11, D-24105 Kiel, Germany

**Keywords:** Topoisomerase II*α*, breast cancer, primary chemotherapy, anthracyclines, Her-2/*neu*

## Abstract

The *α* isoform of Topoisomerase II*α* (Topo IIα) is a proliferation marker as well as a target for several chemotherapeutic agents such as anthracyclines. *In vitro* studies have demonstrated the relationship between the Topo II*α* expression level and chemosensitivity of target cancer cells. To verify this effect *in vivo*, we selected 125 patients presenting with T_2_>3 cm and T_3_ N_0–1_ M_0_ breast tumours who were treated by six cycles of primary chemotherapy, including epirubicin before any surgery. Therapy response was assessed by clinical and X-ray mammogram measurements of tumour shrinkage. The pretherapeutic core biopsies were immunostained with a monoclonal antibody (Ki-S7) against Topo II*α*. Ki-S7 positivity ranged from 0 to 50% (median, 15%). A high percentage of Ki-S7-positive cells (>15%) was associated with tumour regression under chemotherapy (OR=2.88, CI: 1.3–6.4, *P*=0.004). Ki-S7 further emerged as an independent predictor of tumour regression (OR=3.34, CI: 1.41–7.93, *P*=0.006), together with tumour size of less than 40 mm (OR=3.82, CI: 1.58–9.25, *P*=0.002) and negative oestrogen receptor (ER) status (OR=3.35, CI: 1.43–7.86, *P*=0.005), in a multivariate analysis including tumour size, SBR grade, ER and PR status, Ki-67, p53 and Her-2/*neu*. Our clinical results confirm *in vitro* data on the relationship between Topo II*α* expression and tumour chemosensitivity and thus may have important practical implications.

Topoisomerase II*α* (Topo II*α*) is a vital nuclear DNA-binding enzyme that controls and modifies the topologic states of DNA (Berger *et al*, 1996) by combining nuclease, helicase and ligase activities. Topo II*α* reduces DNA supercoiling and twisting by creating a double-strand nick that enables the passage of a second DNA double-strand through the break and subsequent religation of the cleaved DNA strand. Topo II*α* is the specific target of several chemotherapeutic agents, including anthracyclines. These drugs bind to Topo II*α*–DNA complexes and inhibit the religation step, which results in stabilisation of the DNA double-strand breaks that are thought to induce apoptosis (Kellner *et al*, 2002).

It has been previously demonstrated *in vitro* that the sensitivity of tumour cell lines to various Topo II*α* poisons, such as anthracyclines or etoposide, depends on the level of Topo II*α* expression (Gudkov *et al*, 1993; Asano *et al*, 1996a,1996b; Vassetzky *et al*, 1996; Withoff *et al*, 1996a,1996b; Zhou *et al*, 1999; Stacey *et al*, 2000). Moreover, the *TOP2α* gene being located at 17q12–21 close to the Her-2/*neu* oncogene, coamplification of the Her-2/*neu* and *TOP2α* genes was observed in 44% of breast cancers, whereas the *TOP2α* was deleted in another 42% (Jarvinen *et al*, 2000). It also appears that Topo II*α* expression is often correlated to Her-2/*neu* overexpression in breast carcinoma. This complex relationship between the two genes may explain the altered sensitivity to anthracyclines of Her-2/*neu*-amplified breast carcinomas (Bitran *et al*, 1996; MacGrogan *et al*, 1996a; Niskanen *et al*, 1997; Clahsen *et al*, 1998; Jarvinen *et al*, 1998; Paik *et al*, 1998; Thor *et al*, 1998; Vincent-Salomon *et al*, 2000). Most previous studies regarding the effect of Topo II*α* poisons in relation to the cellular level of Topo II*α* expression were either performed *in vitro* or on breast tumour fragments without analysis of the direct *in vivo* effect (Gudkov *et al*, 1993; Asano *et al*, 1996a,1996b; Vassetzky *et al*, 1996; Withoff *et al*, 1996a,1996b; Zhou *et al*, 1999; Stacey *et al*, 2000). The aim of our study was to analyse this effect in a series of breast carcinoma patients treated by primary chemotherapy, and to study the relationship between Topo II*α* and Her-2/*neu* in this setting. To this end, we investigated the predictive and prognostic values of Topo II*α* expression by immunohistochemical detection of the enzyme in breast tumour core biopsies from patients with large operable invasive cancers of the breast treated by primary chemotherapy including epirubicin. Furthermore, we studied the relationship between Topo II*α* expression and different factors modifying tumour chemosensitivity, such as Her-2/*neu*, hormonal receptor, Ki-67 and p53 immunohistochemical detection.

## MATERIALS AND METHODS

### Patients

Immunohistochemical detection of Topo II*α* was performed on tumour core biopsies from 128 patients with primary metastasis-free operable breast cancers, larger than 3 cm. These patients belonged to the neoadjuvant chemotherapy arm of a randomised phase III trial that compared modified radical mastectomy followed by adjuvant chemotherapy to neoadjuvant chemotherapy followed by adapted locoregional treatment in large operable breast tumours. The clinical trial was conducted at Bergonié Institute from January 1985 to April 1989 and included a total of 272 patients. The chemotherapy regimens used in the trial comprised three courses of epirubicin, vincristine and methotrexate (EVM) followed by three courses with mitomycin C, thiotepa and vindesin (MTV), for more details see Mauriac *et al* (1999). All the biopsies analysed in the present study came from the primary chemotherapy, arm of the clinical trial. After completion of the six courses of chemotherapy, clinical examination and mammography were used to assess tumour regression. Subsequent locoregional treatment depended on the extent of tumour regression: radiotherapy was applied exclusively in case of complete regression, conservative surgery with axillary node dissection followed by radiotherapy were performed when tumour regression was incomplete with residual tumour measuring less than 2 cm in diameter; the remaining patients underwent mastectomy.

The predictive and prognostic value of the immunohistochemical detection of oestrogen and progesterone receptors, p53, Her-2/*neu*, and Ki-67 (antibody: Mib-1), had been analysed previously on the same cohort (MacGrogan *et al*, 1996a) and served as reference data for the current study.

### Immunohistochemical assay

The pretherapeutic tumour core biopsies fixed in Holland's fluid and embedded in paraffin were retrieved from the files of Institute Bergonié. Five *μ*m thick were cut, mounted on silane-coated slides and routinely processed. Antigen retrieval was obtained by heating the sections immersed in 0.01 M citrate buffer, pH 6.0, in a pressure cooker for 20 min. The sections were then incubated for 60 min with Ki-S7, a monoclonal antibody specific for Topo II*α*. Ki-S7 was elaborated after immunising Balb/c mice with crude L428 nuclear extracts, performing mouse splenic/myeloma hybridoma cell cultures and purifying cell culture supernatants on a G-Sepharose 4 Fast Flow column (Pharmacia LKB; Freiburg, Germany) (Kellner *et al*, 1997). Ki-S7 specificity for Topo II*α* was verified in immunoprecipitation and Western blot experiments. Ki-S7 immunoreactivity on archival paraffin-embedded tumour material using an antigen retrieval procedure was also controlled (Kellner *et al*, 1997).

Ki-S7 was used undiluted as a lyophilised cell culture supernatant reconstituted with 5 ml H_2_O. The immunoreaction was enhanced by means of an avidin–streptavidin–biotin peroxidase technique (Strept ABC complex/HRP Duet kit, Dako, France) with diaminobenzidine as a chromogen. MCF7 cell pellets with a high percentage of cells in G2 phase were fixed in Holland's fluid and embedded in paraffin to serve as positive controls in each series assayed immunohistochemically for Topo II*α* expression. Negative controls consisted of normal nonhyperplastic epithelial cells present in terminal ductal lobular units in the breast core biopsies.

**Figure 1:**
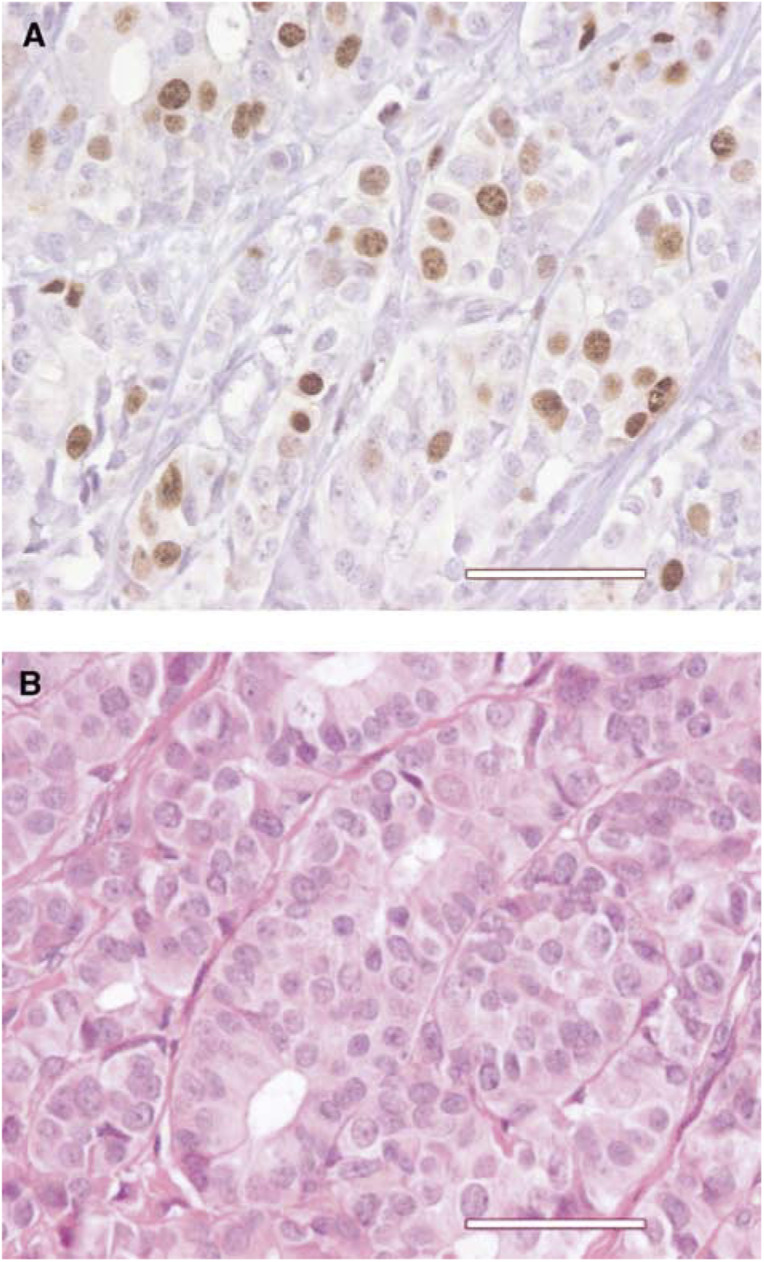
Nuclear immunostaining (Topo II*α*) of 25% cells in a Holland's bouin-fixed paraffin-embedded microbiopsy of an invasive ductal carcinoma NOS using monoclonal antibody Ki-S7 specific for topoisomerase II*α* (**A**). Haematoxylin eosin saffron stain of the same case (**B**). Scale bar=50 *μ*m.

All slides were read by one of the authors (GMG) who was blinded to the clinical results. Only unequivocal nuclear staining of invasive tumour cells was scored as positive (Figure 1

). The percentage of positive tumour cells per tissue section was determined semiquantitatively in 5% steps.

### Statistical analysis

The threshold used for p53 positivity was 1%; for IHC-ER and IHC-PR positivity, the threshold was 10%. These optimal thresholds have already been determined in previous studies to be the most informative for clinical outcome (de Mascarel *et al*, 1995; MacGrogan *et al*, 1995; MacGrogan *et al*, 1996a,1996b). A Ki-67 (Mib1) index of 40% was arbitrarily chosen as a threshold, at the beginning of the study, to differentiate highly proliferating tumours. This Ki-67 threshold had been chosen in our previous study on the same group (MacGrogan *et al*, 1996a), because it corresponded to the 75th percentile of Ki-67 expression in the series. Furthermore, 10% of positive invasive cancer cells with a moderate to strong intensity of staining was chosen for the Her2neu positivity threshold (Slamon *et al*, 2001). Finally, the median values of patients' ages and tumour sizes were chosen as cutoff points for the statistical analysis.

The *χ*^2^ test was used to investigate the significance of the relationship between Ki-S7 and the different immunohistochemical factors previously studied as well as the histological Scarff Bloom and Richardson (SBR) grade. The relation between Ki-S7 and the patient's age as well as tumour size was analysed by Student's *t*-test. A Kendall *τ* rank correlation test was performed to study the relationship between Ki-S7 and Ki-67, considering them as continuous variables.

The clinical size of the tumours was assessed before treatment, before the second and fourth courses of chemotherapy and after the sixth. A univariate analysis studying the relationship between tumour regression and the different factors was performed, using the *χ*^2^ test. Reciprocal influence among the different predictive factors was determined by multivariate analysis using a logistic regression test for multiple proportional hazards. The variable to predict was tumour regression ⩾50%, including complete tumour regression. To evaluate the predictive impact of Ki-S7 expression in this series, a first logistic regression model was performed (model 1), including all previously analysed factors without Ki-S7 (MacGrogan *et al*, 1996a). A second logistic regression model was then performed, after adding Ki-S7 (model 2). All factors were included in the logistic regression analyses, irrespective of their *P*-value by univariate analysis; but only those with a *P*-value ⩽1% were retained in the final models.

The log-rank test using the Kaplan–Meier method was also used to study the relationship between KiS-7 and prognosis expressed as a 5-year probability of survival. Patient follow-up was carried out quarterly for 2 years, twice a year and finally yearly. For overall survival (OS), the survival duration was calculated from the randomisation date to death, or the date they were last known alive. All causes of death were considered as events. For metastasis-free interval (MFI) and disease-free interval (DFI), time to failure was computed from the randomisation date until metastasis or relapse, or the date they were last known to be disease-free, respectively. The median follow-up of patients in this series was 142 months, range 101–172 months.

Univariate and multivariate analyses (logistic regression model) were performed using version 10.0 of SPSS software.

## RESULTS

Invasive carcinoma cells were present in 125 out of 128 biopsies; three core biopsy paraffin blocks contained no residual tumour material after tissue sectioning for histological diagnosis and analysis of the other immunohistochemical factors in the series, and the corresponding cases were excluded from the analysis. Patients' age in this series ranged from 31 to 70 years (mean: 53, median: 55). The mean and median tumour sizes were 43.2 and 40 mm, respectively (range: 20–80 mm). The majority of tumours in this series were invasive ductal carcinomas of no special type (115, 92.2%), followed by invasive lobular carcinomas (9, 7.2%) and one mucous carcinoma (0.6%).

### Ki-S7 expression in the series

Ki-S7 positivity ranged from 0 to 50% with a median value of 15% positive tumour cells. 15% was consequently chosen as the threshold value between low (⩽ 15%) and high (>15%) Topo II*α* expression.

### Relationship between Ki-S7 and other parameters (Table 1)

Ki-S7 was positively associated with SBR grade and p53 expression (*P*=1 × 10^−6^. and 0.03, respectively) and negatively with the immunoexpression of ER and PR (*P*=0.007 and 0.03, respectively). No significant association was found between Ki-S7 and Her-2/*neu* or tumour size (Table 1

**Table 1:** Relationship between Ki-S7 expression and classical and immunohistochemical markers

				**Univariate model**		
**Variables**	**Total^a^**	**No.^b^**	**(%)**	**Odd ratios**	**95% CI^c^**	***P***
Tumour size						
⩽40 mm	69	28	40.6			
>40 mm	55	28	50.9	1.52	0.70–3.31	0.25
Histologic SBR grade						
Grade 1	25	4	16.0			
Grade 2	71	29	40.8	3.63	1.02–14.01	0.024
Grade 3	29	24	82.8	4.87	2.16–11.01	1 × 10^−6^
P53						
0	89	36	40.4			
⩾1%	34	21	61.8	2.38	0.98–5.80	0.034
IHC-ER						
<10%	41	26	63.4			
⩾10%	82	31	37.8	0.35	0.15–0.82	0.007
IHC-PR						
<10%	50	29	58			
⩾10%	71	27	38	0.44	0.20–0.99	0.03
Her2-neu^d^						
Negative	102	46	45.1			
Positive	20	9	45	1.00	0.34–2.87	0.99
KI-67						
⩽40%	97	33	34			
>40%	27	24	88.9	15.52	4.01–70.18	4 × 10^−7^

a All the immunohistochemical analyses for all the markers in the series could not be performed because of the scarcity of tumour material. The number of missing values per marker other than Ki-S7 does not exceed four.

b Number of patients with tumours having >15% KI-S7-positive tumour cells.

c 95% CI: 95% confidence intervals.

d Her2-neu positive cases were those with 10% or more positive tumour cells with a moderate or strong intensity of staining, Her2-neu negative cases were all the other cases.

).

Ki-S7 and Ki-67 were strongly positively correlated (*τ*=0.46, *P*<10^−3^) and associated (*P*=4 × 10^−7^), considering them as continuous or dichotomous variables, respectively.

### Predictive and prognostic values of Ki-S7

High expression of Ki-S7 (>15%) was associated with tumour regression ⩾50% including complete tumour regression after six courses of chemotherapy (OR=2.88, CI: 1.3–6.4, *P*=0.004) by univariate analysis (Table 2

**Table 2:** Factors associated with tumour regression ⩾50%, including complete tumour regression after six cycles of primary chemotherapy among 125 patients: univariate analysis

				**Univariate model**		
**Variables (total)**	**Total**	**No.^a^**		**OR^b^**	**95% CI^c^**	***P***
Age (years)						
⩽55	65	32				
>55	60	23		0.64	0.30–1.39	0.220
Clinical tumour size						
>40 mm	55	17				
⩽40 mm	69	37		2.58	1.16–5.83	0.011
Histologic SBR grade						
Grade 1	25	9				
Grade 2	71	29		1.23	0.43–3.51	0.67
Grade 3	29	17		2.52	0.73–8.85	0.09
ER						
⩾10%	82	26				
<10%	41	27		4.15	1.75–9.99	<10^−3^
PR						
⩾10%	71	27				
<10%	50	25		1.63	0.73–3.63	0.190
Her2-neu^d^						
Negative	102	41				
Positive	20	11		1.82	0.63–5.30	0.221
Ki-67						
⩽40%	97	36				
>40%	27	18		3.39	1.27–9.21	0.006
Ki-S7						
⩽15%	68	22				
>15%	57	33		2.88	1.3–6.4	0.004
P53						
0	89	36				
⩾1%	34	17		1.47	0.62–3.51	0.339

a #:number of patients with tumour regression ⩾50%, including complete tumour regression after six cycles of primary chemotherapy.

b OR=odd ratios.

c 95CI=95% confidence interval.

d Her2-neu positive cases were those with 10% or more positive tumour cells with a moderate or strong intensity of staining, Her2-neu negative cases were all the other cases.

). The median Ki-S7 value was higher in the good response group (tumour regression ⩾50% and complete tumour regression) compared to the poor response group (tumour regression <50%, tumour stabilisation and progression), 20 and 15%, respectively. To evaluate the predictive impact of Ki-S7 in this series, a first stepwise logistic regression model was performed with all the factors that had been previously analysed in the series, that is, clinical tumour size, SBR grade, IHC-ER, IHC-PR, Ki-67, p53 and Her-2/*neu* (Table 3

**Table 3:** Factors associated with tumour regression ⩾50%, including complete tumour regression after six cycles of primary chemotherapy among 125 patients: multivariate analyses (model 1: factors identified in MacGrogan 1996a; model 2: with new factor, Ki-S7)

	**Model 1**					**Model 2**				
	**B**	**Standard error**	**OR**	**95 CI**	***P***	**B**	**Standard error**	**OR**	**95 CI**	***P***
Clinical tumour size										
>40 mm		Reference					Reference			
⩽40 mm	1.327	0.451	3.771	1.55–9.12	0.003	1.342	0.450	3.82	1.58–9.25	0.002
IHC-ER										
⩾10%		Reference					Reference			
<10%	1.212	0.436	3.362	1.43–7.9	0.005	1.210	0.435	3.35	1.43–7.86	0.005
Ki-67										
⩽40%		Reference					Excluded from model			0.092
>40%	1.364	0.528	3.910	1.39–10.99	0.010					
Ki-S7										
⩽15%	–	–	–	–	–		Reference			
>15%	–	–	–	–	–	1.208	0.441	3.347	1.41–7.93	0.006

Each of the odds ratios is adjusted for all other variables in the table. OR=odd ratio. 95 CI=95% confidence interval.

, model 1). In this model, clinical tumour size less than 40 mm, negative IHC-ER status and high expression of Ki-67 (>40%) were found to be independent predictive factors for tumour regression. When Ki-S7 was added (Table 3, model 2), independent predictive factors were clinical tumour size, IHC-ER and Ki-S7. Ki-67 expression was no longer considered an independent predictive factor for tumour regression in this second model (*P*=0.092).

Considering the impact of concurrent Topo II*α* and Her-2/*neu* overexpression on tumour chemosensitivity described in the literature, the association of these two proteins was analysed in relation to tumour regression, but no significant correlation was found either by univariate or multivariate analysis.

By univariate analysis, no statistically significant relationship was found between Ki-S7 and DFI, MFI or OS (results not shown).

## DISCUSSION

Previous studies, showing a relationship between the intracellular level of Topo II*α* and tumour chemosensitivity, were mainly based on *in vitro* experiments analysing the efficacy of other Topo II*α* inhibitors in various tumour cell lines, in particular etoposide (Gudkov *et al*, 1993; Asano *et al*, 1996a,1996b; Vassetzky *et al*, 1996; Withoff *et al*, 1996a,1996b; Stacey *et al*, 2000). In the current study, we have demonstrated for the first time a significant direct *in vivo* correlation between the level of Topo II*α* expression and breast cancer response to anthracycline-based chemotherapy. This result argues in favour of the concept that the efficacy of Topo II*α* poisons depends on the quantity of stabilised cleavable complexes, which in turn relates to the cellular expression and activity of Topo II*α* (Kellner *et al*, 2002).

Jarvinen *et al*, (1998) found no significant correlation between Topo II*α* expression in primary breast tumours and the regression of metastases after chemotherapy in patients with advanced breast cancer treated with first-line epirubicin. The discrepancy between their results and ours may be attributable to different Topo II*α* contents in primary tumours and their metachronous metastases Indeed, in a series of 13 breast tumours, the same group subsequently showed some degree of variability between the Topo II*α* gene status of the primary breast tumour and that of its metastases by means of fluorescent *in situ* hybridisation (FISH) and chromogenic *in situ* hybridisation (CISH) (Tanner *et al*, 2001). Finally it is conceivable that due to intervening acquisition of drug-resistance mechanisms (Giesler *et al*, 2002), the effect of Topo II*α* inhibitors may be different in primary tumours and their corresponding asynchronous metastases, especially if an adjuvant protocol comprising a Topo II*α* inhibitor had previously been applied.

In line with the proliferation-specific expression of Topo II*α* (Kellner *et al*, 1997,^2002^), we observed a highly significant correlation between Ki-S7 and Ki-67, which was also reported by others (Depowski *et al*, 2000; Rudolph *et al*, 1999a; Tuccari *et al*, 1993), in addition to a strong link between the number of Topo II*α*-expressing cells and the S-phase fraction of the cell cycle (Jarvinen *et al*, 1996; Sandri *et al*, 1996a). Given the known association between high tumour cell proliferation and chemosensitivity (Tannock, 1978; Belembaogo *et al*, 1992; O'Reilly *et al*, 1992), one might suspect the observed therapy response to be related to the proliferative activity of the tumours. Indeed, when we previously investigated the predictive value of various immunohistochemical factors in the same cohort (MacGrogan *et al*, 1996a), Ki-67 came out as an independent predictive factor for the therapy response in the multivariate analysis. After inclusion of Ki-S7 into the logistic regression model, however, Ki-67 lost its statistical significance. This suggests that Topo II*α* expression may have an impact on the responsiveness of tumours to cytotoxic therapy beyond its association with tumour cell proliferation, which is likely related to an increased sensitivity to the Topo II*α* inhibitory component of the treatment protocol, that is, epirubicin (Kellner *et al*, 2002). It is not possible to reproduce *in vitro* experiments identically *in vivo* because no current therapy protocol for breast cancer consists of a monotherapy with Topo IIα inhibitors. It would nevertheless be difficult to explain the predictive superiority of Ki-S7 compared with Ki-67 than by a higher availability of substrate for the anthracycline component of the therapy regimen (Kellner *et al*, 2002). It is true that the assessment of the immunopositive cell fraction does not allow conclusions as to the protein content of individual cells, and that a low expression at the cellular level might account for the failure of a number of tumours with a high percentage of Ki-S7-positive cells to respond to therapy. More likely, however, this lack of responsiveness is attributable to resistance mechanisms (Kellner *et al*, 2002) acquired during tumour progression, consistent with a lower response rate in larger tumours (Gieseler *et al*, 2003).

On the other hand, we found a high Ki-S7 expression indicative of an aggressive tumour phenotype in terms of higher SBR grade, hormonal receptor negativity and p53 overexpression. These observations substantiate the results of previous studies (Jarvinen *et al*, 1996; Rudolph *et al*, 1999a,1999b). Since wild-type p53 negatively regulates the promoter of the human *TOP2α* gene (Sandri *et al*, 1996b), the immunohistochemical detection of p53, consistent with a nonfunctional protein, may be expected to be associated with high levels of Topo II*α*.

Contrary to previous results from our group (Rudolph *et al*, 1999a) and others (Jarvinen *et al*, 1996,^1998^; Rudolph *et al*, 1999b), we failed to observe a significant positive correlation between Topo II*α* expression and Her-2/*neu*-overexpression in this series. Approximately 50% of the Her-2/*neu* overexpressing tumours in our series contained high levels of Topo II*α*, whereas in the remaining 50%, Topo II*α* was low (Table 1). This finding is congruent with the results of a study by Järvinen *et al* (2000). In 97 breast cancers, these authors found no Topo II*α* gene copy aberrations when the Her-2/*neu* gene status was normal, yet when Her-2/*neu* was amplified, *TOP2α* was coamplifed in 44% and deleted in 42% of the cases. Such data may explain the contradictory results of different reports analysing breast cancer susceptibility to Topo II*α* inhibitors in relation to the Her-2/*neu* status (Muss *et al*, 1994; Paik *et al*, 1998; Vincent-Salomon *et al*, 2000). In the same study, Järvinen *et al* (2000) observed an increased sensitivity to doxorubicin in a breast cancer cell line (UACC-812) with *TOP2α* gene amplification and consequent protein overexpression and conversely, a decreased sensitivity to doxorubicin in a breast cancer cell line with *TOP2α* deletion and concomitantly reduced protein content (MDA-361). In a previous study on 863 primary operable invasive ductal carcinomas with long-term follow-up (Rudolph *et al*, 1999a), of which only a minor proportion had been treated with Topo II*α* inhibitors, high Topo II*α* expression consequently emerged as an important independent predictor of adverse outcome next to nodal metastasis and before other classical or immunohistochemical factors, including Ki-67. However, no such correlation between Topo II*α* and survival was found in the present series. This could be attributable to a balanced effect of the Topo II*α* expression portending increased chemosensitivity to Topo II*α* inhibitors on the one hand and a higher malignant potential on the other (Kellner *et al*, 2002).

Primary chemotherapy in breast cancer enhances the rate of breast conservation and enables the *in vivo* assessment of tumour sensitivity to different chemotherapeutic drugs. Clinical decisions concerning the choice of cytotoxic therapy regimens might be improved according to the initial tumour response (Wolff and Davidson, 2000). With this in view, it would be advantageous to be able to reliably estimate tumour pathologic and biologic features beforehand in order to tailor the composition of chemotherapeutic protocols. Mindful of the fact that anthracyclines target Topo II*α*, we have shown that tumours expressing high levels of Topo II*α* responded better to an anthracycline-based cytotoxic protocol than tumours with low Topo II*α* expression. Therefore, we conclude that Topo II*α* assessment may be of value for the clinical management of breast cancer in the setting of primary chemotherapy, provided that our results are corroborated by further studies.
